# Author Correction: Establishing coherent momentum-space electronic states in locally ordered materials

**DOI:** 10.1038/s41467-024-54127-y

**Published:** 2024-11-08

**Authors:** Samuel T. Ciocys, Quentin Marsal, Paul Corbae, Daniel Varjas, Ellis Kennedy, Mary Scott, Frances Hellman, Adolfo G. Grushin, Alessandra Lanzara

**Affiliations:** 1 Department of Physics, University of California, Berkeley, CA 94720 USA; 2 Materials Science Division, Lawrence Berkeley National Laboratory, Berkeley, CA 94720 USA; 3 Univ. Grenoble Alpes, CNRS, Grenoble INP, Institut Néel, 38000 Grenoble, France; 4 Department of Physics and Astronomy, Uppsala University, Box 516, 751 20 Uppsala, Sweden; 5 Department of Materials Science, University of California, Berkeley, CA 94720 USA; 6 Department of Physics, Stockholm University, AlbaNova University Center, 114 21 Stockholm, Sweden; 7 The Max Planck Institute for the Physics of Complex Systems, 01187 Dresden, Germany; 8 Department of Theoretical Physics, Institute of Physics, Budapest University of Technology and Economics, Műegyetem rkp. 3., H-1111, Budapest, Hungary; 9 IFW Dresden and Würzburg-Dresden Cluster of Excellence ct.qmat, Helmholtzstrasse 20, 01069 Dresden, Germany; 10 Molecular Foundry, Lawrence Berkeley National Laboratory, Berkeley, CA 94720 USA

**Keywords:** Electronic properties and materials, Materials for devices, Surfaces, interfaces and thin films

Correction to: *Nature Communications* 10.1038/s41467-024-51953-y, published online 17 September 2024

The original version of this article contained an error in Fig. 2, where panel (e) displayed incorrect data and had an incorrect label on the *y*-axis. The correct version of Fig. 2 is:


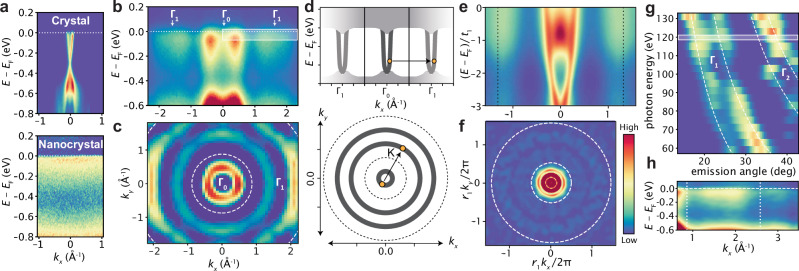


which replaces the previous incorrect version:
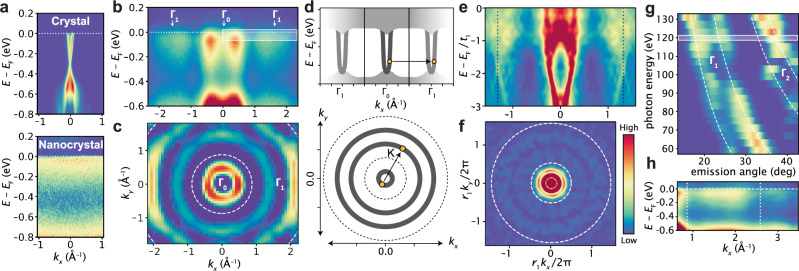


This has been corrected in both the PDF and HTML versions of the Article.

