# Supplementary material for: Establishing coherent momentum-space electronic states in locally ordered materials
Source: Nat Commun. 2024 Sep 17;15:8141. doi: 10.1038/s41467-024-51953-y (PMC11408612; doi:10.1038/s41467-024-51953-y)
Supplement: Supplementary file 1 — Supplementary Information [file 41467_2024_51953_MOESM1_ESM.pdf]

Supplementary Information:
Establishing Coherent Momentum-Space Electronic States in Locally Ordered Materials

Samuel T. Ciocys,[1,2] Quentin Marsal,[6] Paul Corbae,[2,3] Daniel Varjas,[7] Ellis Kennedy,[3,4]
Mary Scott,[1,5] Frances Hellman,[1,2] Adolfo G. Grushin,[6] and Alessandra Lanzara[1,2*]

[1]Department of Physics, University of California,
Berkeley, California, 94720, USA

[2]Materials Science Division, Lawrence Berkeley National Laboratory,
Berkeley, California, 94720, USA

[3]Department of Materials Science, University of California,
Berkeley, California, 94720, USA

[4] National Center for Electron Microscopy,
Molecular Foundry, Lawrence Berkeley National Laboratory,
Berkeley, California, 94720, USA

[5] Molecular Foundry, Lawrence Berkeley National Laboratory,
Berkeley, California, 94720, USA

[6]Univ. Grenoble Alpes, CNRS, Grenoble INP, Institut Néel,
38000 Grenoble, France

[7]Department of Physics, Stockholm University, AlbaNova University Center,
106 91 Stockholm, Sweden

[*]To whom correspondence should be addressed; E-mail: alanzara@lbl.gov

(Dated: July 25, 2024)

Supplementary Note 1: Overview

Amorphous matter has been historically categorized as being localized, and often wrongly concluded to be nondispersive [1]. Our work shows that this long standing assumption is false: there can be dispersive, coherent electronic states in a locally ordered amorphous material which has no long range order. We include this summary to link together the pieces of our experiment to paint the clearest picture of our results and novelty.

Main Figure 1 firstly highlights the well defined local, short-range order in real space leads to a defined reciprocal space even in the case of no long range order. It also highlights that random ordering does not give a momentum space structure, which is why amorphous materials have been often disregarded as having no momentum space structure. Main Figure 2 presents our main experimental result, that there are dispersive features crossing the Fermi level and these features are repeated in momentum space. These results experimentally prove that the widespread notion that amorphous materials should be featureless is misguided. The experimental data is complemented by a model that accurately describes our amorphous system and reproduces the vertical, dispersive features as well as the replication at higher momenta. Beyond the approximations, the theoretical result that repetitions will be present if local order exists is a big conceptual leap, especially for condensed matter physicist working with crystalline lattices. We show for the first time that ARPES is a tool which elucidates how their existing order is imprinted in their momentum-resolved electronic structure. Main Figure 3 gives us a comparison of the crystalline and amorphous electronic states, especially the bulk ones stemming from a similar local order between the two. It also helps the reader to understand the photoemission process in an amorphous material. Finally, in Main Figure 4 we show how a Dirac dispersion would behave in an amorphous system, becoming more vertical and broad, in good agreement with our experimental ARPES data. This type of renormalization of the spectrum, rooted in the structure factor of the amorphous atomic network, has not been previously predicted or observed. Taken all together, we show that local order leads to momentum space structure and dispersion in an amorphous material. The delocalized, dispersive character of the surface states in the system lead to coherent states beyond multiple neighboring atomic sites.

Main Figure 1 is complemented by Supplementary Notes 2, 3, 4, 5, which provide details about the sample growth process and microscopic structure. The raw data from the experiment and the simulations that were used to plot Main Figure 2 are showed in Supplementary Note 6. Moreover, to illustrate the generality of our conclusions, in Supplementary Note 7 we also study the case of monolayer amorphous carbon, that shares the same local order as graphene. Further discussions about the photon-energy dependence of the amorphous samples can also be found in Supplementary Note 8. The details of the calculation in Main Figure 4 are presented in Supplementary Note 9.

Supplementary Note 2: Thin film growth

The Bi_2Se_3 films used for this study were grown on 300 nm of amorphous SiN_x on top of 500 micron Si(100) (10x10x0.5mm^3) using a standard thermal evaporation technique. A custom built UHV chamber with base pressure

of 10^{-9} Torr was used to grow the films. The films were co-deposited from high purity (99.999%) elemental Bi and Se single sources. The films were grown at room temperature with fluxes to match the stoichiometric ratio. The films were capped with 50nm of Se to prevent atmospheric exposure.

Supplementary Note 3: MD

Density functional theory (DFT) calculations were performed using the projector augmented wave (PAW) formalism in the Vienna ab initio Simulation Package (VASP) [2, 3]. The exchange-correlation potentials were treated in the framework of generalized gradient approximation (GGA) of Perdew-Burke-Ernzerbof (PBE) [4]. Bi (6s, 6p) and Se(4s, 4p) electrons were treated as valence, and their wavefunctions expanded in plane waves to an energy cutoff of 500 eV. A k-point grid of 3x3x1 with Gamma sampling was used. Spin-orbit coupling was added self-consistently for all density of states calculations. Amorphous structures were generated with ab initio molecular dynamics using VASP.

Supplementary Note 4: RDF

High resolution TEM and Fluctuation electron microscopy (FEM) were performed on 10 nm thick Bi_2Se_3 films deposited on a 10 nm thick SiN window. Diffraction images were collected on an Orius CCD system with an exposure time of 0.3 seconds and a camera length of 300 mm. The central beam was covered using a beam stop. Imaging conditions were held constant for all data collection to prevent variations in microscope alignment. The RDF was computed by masking beam stop, computing the radially integrated intensity, computing the structure factor after subtracting off the single atom scattering, the FFT to get the RDF.

Supplementary Note 5: Ruling Out Selenium's Role on Surface State

We performed glancing angle XPS to rule out that the observed surface states are due to a thin remaining layer of selenium atoms following the selenium decapping process. If excess selenium were present, then the XPS signal from the Se core levels would be enhanced as the angle of incidence relative to the sample surface normal is increased. Supplementary Figure 1 shows near identical spectra between glancing and near normal XPS. Therefore, the stoichiometry of the surface is very similar to the bulk.

Moreover, when the samples are grown non-stoichiometrically (see Supplementary Figure 2) with increased selenium, the coherent band structure is lost.

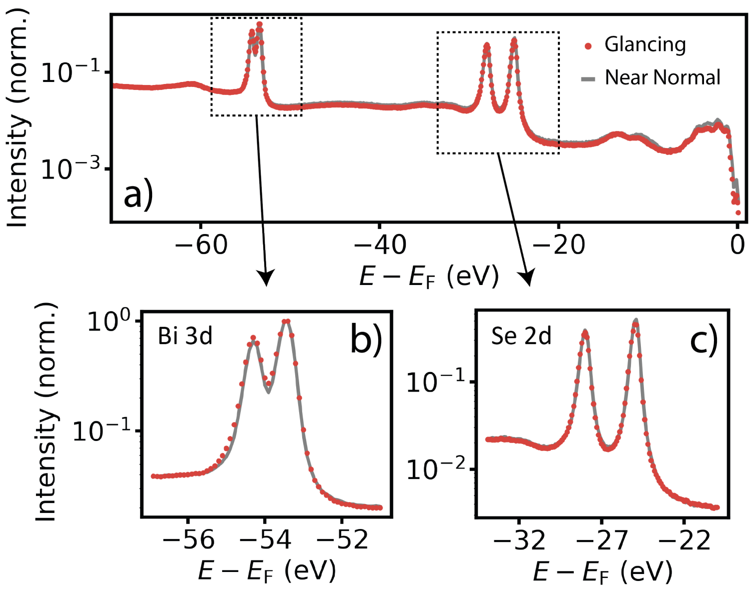

SUPP. FIG. 1. **Glancing Incidence XPS on Se and Bi Core Levels. (a)** XPS performed at $h\nu = 220$ eV displaying the Bi 3d and Se 2d core level peaks as well as the valence features near $E - E_F = 0$. Near-normal emission (grey) and glancing incidence (red) show nearly identical spectra. **(b,c)** Zoom in of Bi and Se core levels demonstrating that emission stoichiometry is equivalent, meaning Se is not more abundant at the surface than the bulk.

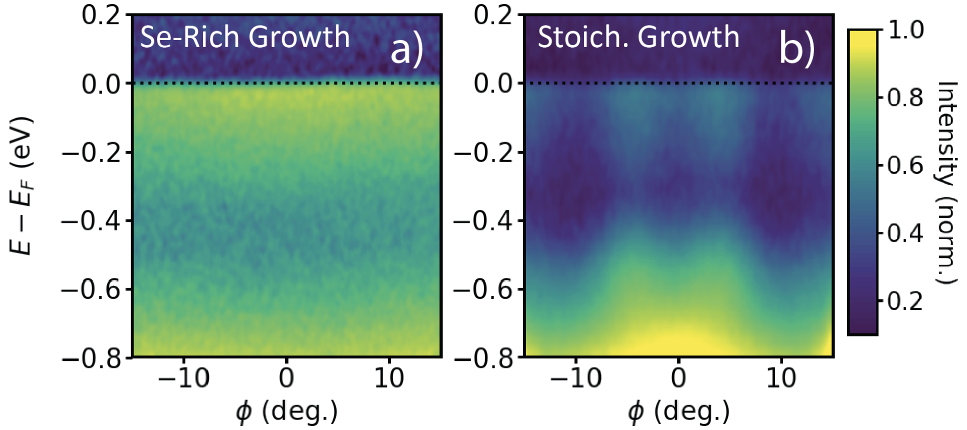

SUPP. FIG. 2. **ARPES Comparison of Stoichiometric and Se-Heavy Growth. (a)** Selenium-rich growth produces no momentum-dependnent band structure. **(b)** Stoichiometric Bi_2Se_3 amorphous growth leads to the observed surface state vertical features.

Supplementary Note 6: Raw ARPES Data of Amorphous Fermi Surface

The Fermi surface shown in Main Figure 2(c) is derived from the Fermi surface in Supplementary Figure 3 seen above. The main figure was produced by symmetrizing the above spectrum across $k_x = 0$ and performing second derivatives along both the k_x and k_y directions in order to more clearly observe the ring structures in the second Brillouin-like zone (BLZ). However, the ring like structures both in the first BLZ and second BLZ are apparent in the raw Fermi surface as well with clear radial symmetry.

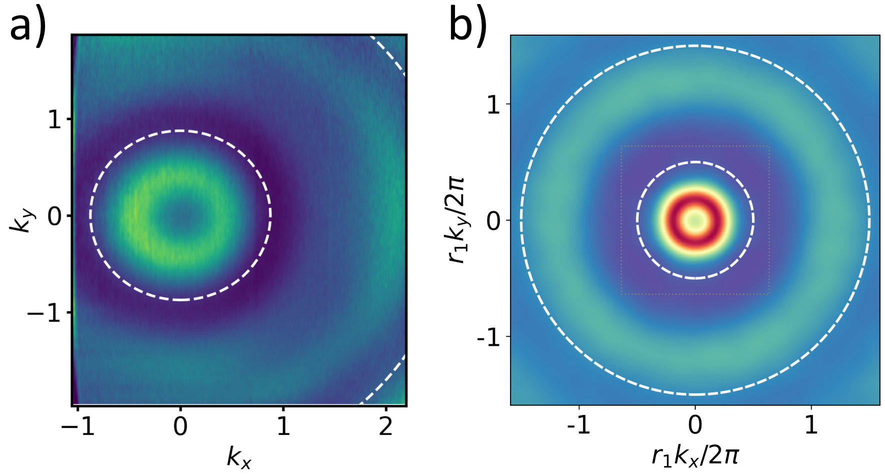

SUPP. FIG. 3. (a) Raw Fermi Surface, measured by the experiment. (b) Spectral function at a constant energy, the Laplacian of which is shown in Main Figure 2(c).

Supplementary Note 7: Monolayer Amorphous Carbon

To illustrate the interplay between the spatial arrangement of atoms and the momentum spectrum of the system, we compute the ARPES response of a simpler, two-dimensional amorphous system: monolayer amorphous carbon [5].

Transmission electronic microscopy data show that monolayer amorphous carbon is organized into small hexagonal graphene-like clusters oriented in random directions and separated by regions of random filling where carbon atoms organize in pentagons or heptagons while their coordination remains fixed to three [5]. To control the degree of crystallinity, we introduce the fraction of hexagons over the total number of polygons formed by carbon atoms f_6. We study three lattice realizations with a different degree of crytallinity: perfect graphene ($f_6 = 1$, Supplementary Figure 4(a)), polycrystalline graphene ($f_6 = 0.76$, Supplementary Figure 4(b)) and monolayer amorphous carbon ($f_6 = 0.55$, Supplementary Figure 4(c)). Monolayer amorphous carbon can be seen as the amorphous counterpart of graphene. All three realizations are modelled it with a tight-binding model with a constant hopping on each bond: $H_{MAC} = \sum_{\langle i,j \rangle} t \hat{c}_i^\dagger \hat{c}_j + \text{h.c.}$, where $\langle \cdots \rangle$ indicates nearest neighbours.

The three lattice realizations can be characterized by their structure factor, shown in Supplementary Figure 4(d, e, f). The structure factor is the spatial Fourier transform of the lattice site positions. The k-scale will be explained shortly. The structure factor of the perfect crystalline lattice is periodic in momentum space (Supplementary Figure 4(d)). The period of the honeycomb lattice is set by a, the lattice constant of each triangular sublattice. It determines the periodicity of the structure factor as $k_{\text{cryst}} = \frac{2\pi}{a}$.

The structure factor of the polycrystalline lattice (Supplementary Figure 4(e)) shows a ring-like structure centered around the Γ point. It is not completely isotropic since the intensity varies along the rings. In fact, the structure factor of the polycrystalline lattice can be interpreted as the superposition of the structure factor of each crystallite, with random orientations.

The structure factor of the monolayer amorphous carbon is fully isotropic. It also shows rings but not as many as

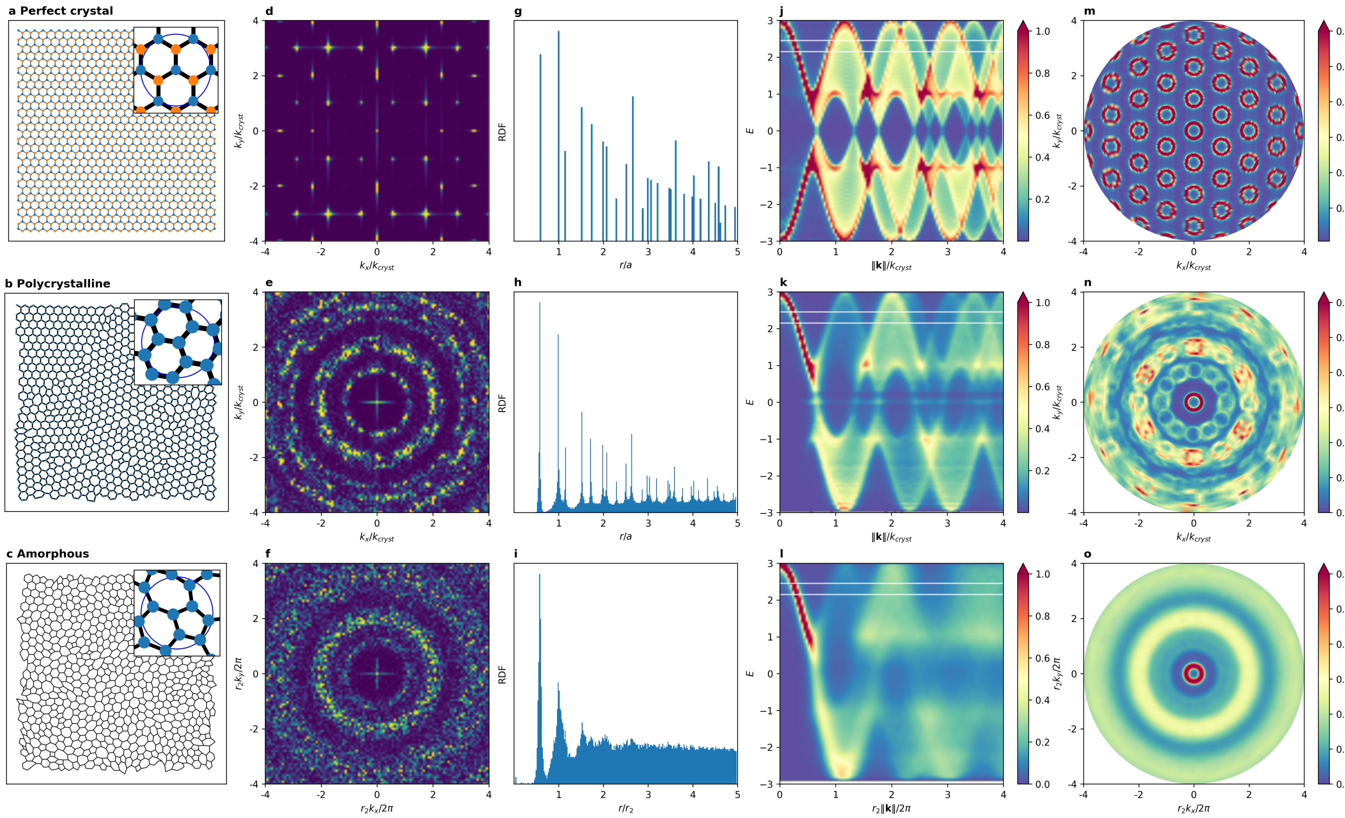

SUPP. FIG. 4. **Comparison between crystalline, crystalline and amorphous graphene.** The first column (**a, b, c** shows the lattice structure for three different systems: crystalline graphene (**a**), polycrystalline graphene (**b**) and monolayer amorphous carbon (**c**). The insets show the typical local environment of a site. The radius of the blue circle in the insets is a for lattices **a** and **b** and r_2 for lattice **c**. The second column (**d, e, f**) shows the corresponding structure factor as a function of the wave vector $\mathbf{k}$, in units of $k_{\text{cryst}} = \frac{2\pi}{a}$ for **d** (lattice **a**) and **e** (lattice **b**), and $k_2 = \frac{2\pi}{r_2}$ for **f** (lattice **c**). The third column (**g, h, i**) shows the radial density function (RDF) of each system. The period a of crystalline graphene corresponds to the second-nearest-neighbour distance in the honeycomb lattice. In lattice **c**, we use the averaged second-nearest-neighbour distance r_2 as the unit length in order to have comparable results with lattices **a** and **b**. The fourth column (**j, k, l**) shows the ARPES spectrum of each system realization averaged over the in-plane angle. The three spectra look similar in the first Brillouin zone but the fluctuations in bond length tend to blur them at large $\|\mathbf{k}\|$. Near the Γ point, there are no states at negative energy in the disordered samples since polygons with an odd number of edges forbid such a state. The fifth column (**m, n, o**) shows a constant-energy cut of the ARPES spectra drawn at $E = 1.2t$ (white area in **j, k, l**). The graphene spectrum is periodic in $\mathbf{k}$. The random orientation of the bonds between crystallites in polycrystalline graphene (**b**) or at local scale in monolayer amorphous carbon (**c**) results in an isotropic spectra. In panels **l, o**, the response of the amorphous material is averaged over 20 disorder realizations. The third and fourth columns show that the response of monolayer amorphous graphene at small $\mathbf{k}$ can be seen as a rotation-averaged equivalent of that of graphene.

the polycrystalline graphene because of the disorder in the arrangement of the atoms. At large scales (low momenta), the structure factor still depends on $\|\mathbf{k}\|$. This shows that the position of the sites is not completely uncorrelated. The fluctuations in the nearest-neighbour distances are low enough to average out at large scales. Monolayer amorphous carbon is therefore a hyperuniform system [6].

Focusing on position space, the radial density function (RDF, Supplementary Figure 4(g, h, i) gives information about the average distance between neighbouring sites. It is proportional to the density of neighbouring sites as a function of their relative distance. In crystals, atoms are regularly spaced. The RDF **g** thus shows sharp peaks at the nearest-neighbour distance, second-nearest-neighbour distance, *etc.* Since graphene forms a honeycomb lattice, the

crystal period a equals the second-nearest-neighour distance.

In polycrystalline graphene, each crystallite remains perfect but disorder appears due to their different orientations. The RDF **h** shows broadened peaks centered at the same positions as those of the crystal RDF. Indeed, the period of each crystallite a still corresponds to the second-nearest-neighbour distance and may still be used to compare the scales of systems **a** and **b**.

In monolayer amorphous graphene, the arrangement of atoms is disordered but the fluctuations in the nearest-neighbour distances remain under control. The lattice is disordered at large scale but the local environment of each site remains approximately the same. The RDF **i** shows only a few broadened peaks. Disorder dominates at distances comparable to the third or fourth neighbour. The second-nearest-neighbour peak being still visible, indicates that we can use the averaged second-nearest-neighbour distance r_2 to set the scale of the plots in the third row both in position space and momentum space. These results is consistent with the measurement of the distribution of nearest-neighbour distances and bond angles of real monolayer amorphous carbon [5]. Both are broader than in graphene but remain centered around the same average values because the geometry of the lattice at short scales is constrained by the chemical characteristics of carbon atoms.

One can use these three lattice realizations with increasing disorder to compare their ARPES response and understand how it depends on the arrangement of the atoms. For each of the lattice realizations, we thus compute the spectral function of the lattice, which is proportional to the ARPES response. We obtain the spectral function using the kernel polynomial method [7], with 200 moments, and average over 20 disorder realization for the amorphous case. The fourth and fifth columns of Supplementary Figure 4 show the rotation-averaged spectral function as a function of $\|\mathbf{k}\|$ (panels **j** to **l**) and a constant energy cut of the same spectrum as a function of $\mathbf{k}$ for the three systems (panels **m** to **o**).

The spectrum of graphene (panels **j** and **m**) is periodic in momentum with period $\frac{2\pi}{a}$. As a function of energy, it exhibits Dirac cones around the K points in each unit cell of the Bravais lattice.

The spectrum of polycrystalline graphene (panels **k** and **n**) is not periodic, but the first Brillouin zone seems unchanged compared to monocrystalline graphene, at least for positive energy. Indeed, the states with $\mathbf{k} = 0$ and negative energy are completely antisymmetric with respect to bond inversion. Thus they require the existence of well defined sublattices, which is impossible if the lattice contains loops with an odd number of edges. Apart from this, the spectra **j** of **a** and **k** of **b** are very similar, and that column shows that the spectrum of polycrystalline graphene can be interpreted as the rotation average of that of monocrystalline graphene, due to the random orientation of the crystallites.

The spectrum of monolayer amorphous carbon (panels **l** and **o**) is also isotropic, and similar to the rotation-averaged crystalline spectrum. However, the rings blur at lower momenta than in the polycrystalline spectrum, indicating that disorder plays a prominent role at longer length scales. One can quantify the strength of disorder by measuring the half-height width of the first peak in the radial density function δr. In the monolayer amorphous carbon (panel **i**), one can measure $\delta r \approx 0.20 - 0.25\ r_2$. As a consequence, for $\|\mathbf{k}\| \sim 2\pi/\delta r$ the fluctuations in the

nearest-neighbour distance are of the same magnitude as the measured wavelength of the electrons. At this scale, the phase shift between neighbouring sites becomes uncorrelated and the ARPES response blurs. Therefore, the rings in the monolayer amorphous carbon ARPES response are visible up to $\|\mathbf{k}\| \approx 4 - 5\ \frac{2\pi}{r_2}$. This limit approximately corresponds to what can be seen on Supplementary Figure 4(l).

At large length scales, i.e. momenta around the Γ point, the ARPES response does not depend on the strenght of disorder. In this regime, the three lattices can be understood as continuous systems [8, 9], therefore the exact position of the sites is not relevant. When the measured wavelength shortens, or the momentum increases, the responses differ much more. The crystalline ARPES is periodic in momentum since it reflects the translation symmetry of the lattice whereas both the polycrystalline and amorphous responses tend to be isotropic due to the random orientation of the bonds. In non-periodic systems, when the wavelength becomes smaller than the typical fluctuations in the bond lengths, the ring structure blurs. These fluctuations are very low in polycrystalline systems, thus allowing to see rings up to relatively high momenta. They are inherent to amorphous lattices, so one can only see the rings corresponding to the few first Brillouin zones in the ARPES response of such systems.

Supplementary Note 8: Photon Energy Dependence of Vertical Features

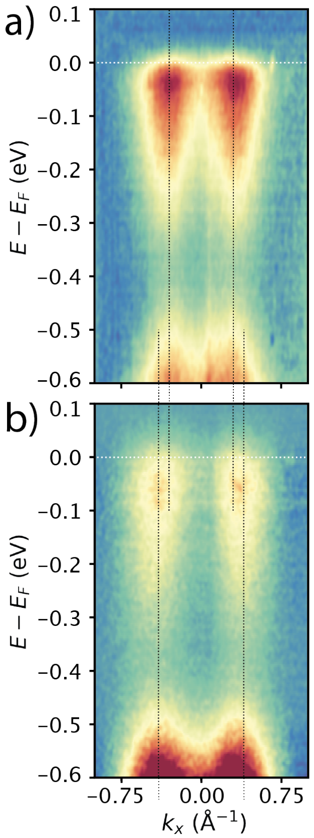

SUPP. FIG. 5. **Photon-energy dependence of k_x-scaling (a)** Surface state spectrum at $h\nu = 60$ eV with vertical features centered at ±0.30 Å^{-1}. **(b)** Surface state spectrum at $h\nu = 60$ eV with vertical features centered at ±0.41 Å^{-1}. k_x rescaling is seen both near E_F and near the valence band.

Supplementary Note 9: Scattering of quasi-free 2D Dirac electrons

In the main text, we consider the scattering of the surface state on atomic centers as a possible explanation for the discrepancies between the crystalline and the amorphous surface-state dispersion relations. In this section, we extend the method proposed in [10, 11] to the full Born approximation, and generalize it to quasi-free Dirac electrons at the surface of a crystalline or amorphous topological insulator.

The effect of scattering on atoms on the quadratic dispersion relation of almost free electrons was studied, in the first Born approximation, in [10, 11]. The scattering effect due to the atomic, crystalline or amorphous, lattice is treated as a perturbation compared to a continuum medium with a uniform background. For electrons with a quadratic dispersion relation, the Hamiltonian thus reads [10]

$$\hat{H} = \frac{\hat{p}^2}{2m} + \langle\hat{V}\rangle + \hat{V} - \langle\hat{V}\rangle = \hat{H}_0 + \hat{H}_V, \tag{1}$$

where $\hat{V} = \sum_i^N \hat{U}(\mathbf{r} - \mathbf{r}_i)$ is the scattering landscape created by the atomic centers, and $\langle\hat{V}\rangle = N\langle\hat{U}\rangle$ is the corresponding uniform average background.

The perturbation $\hat{H}_V = \hat{V} - \langle\hat{V}\rangle$ introduces a self-energy correction to the Green's function, which is given in the first Born approximation by

$$\hat{\Sigma}(E, \mathbf{k}) = \frac{1}{\Omega}\int c_2(\mathbf{k} - \mathbf{k}')|v(\mathbf{k} - \mathbf{k}')|^2 \hat{G}_0(\mathbf{k}')\mathrm{d}\mathbf{k}', \tag{2}$$

where Ω is the surface area of the system, $v(\mathbf{k})$ is the Fourier transform of $\hat{U} - \langle\hat{U}\rangle$, and disorder enters through the structure factor of the amorphous lattice $c_2(\mathbf{k}) = \sum_{i,j} e^{i\mathbf{k}\cdot(\mathbf{r}_i - \mathbf{r}_j)}$. For a point-like interaction $\hat{U}(\mathbf{r}) \propto \delta(\mathbf{r})$, $v(\mathbf{k} - \mathbf{k}') = v_0$ is uniform in momentum space.

In the full Born approximation, Eq. (2) becomes a self-consistent equation,

$$\hat{\Sigma}(E, \mathbf{k}) = \frac{v_0^2}{\Omega}\int c_2(\mathbf{k} - \mathbf{k}')\hat{G}(E, \mathbf{k}')\mathrm{d}\mathbf{k}', \tag{3}$$

where $\hat{G} = (E - \hat{H}_0 - \hat{\Sigma})^{-1}$. Numerically, this integral is sampled with finite resolution δk and computed using a fast Fourier transform. Thus, the densities of states plotted in Supplementary Figure 6, and Main Figure 4 of the main text are obtained from solutions of an equation of the form

$$\Sigma(\hat{E}, \mathbf{k}) = A\sum_{\mathbf{k}'} \frac{\tilde{c}_2(\mathbf{k} - \mathbf{k}')}{E - \hat{H}_0 - \alpha\Sigma(E, \mathbf{k})}, \tag{4}$$

where $A = \frac{v_0^2 N_{\text{atoms}}\delta k}{\Omega}$, $\tilde{c}_2 = \frac{c_2}{N_{\text{atoms}}}$ is the renormalized structure factor, Ω is the surface of the system, and $\alpha \sim 10^{-1}$ is coefficient that we introduced to improve the numerical stability of the self-consistent solution, assuming that the self-energy is proportional to A. In our calculations, we chose $a\delta k = 0.03$ and $A \sim 10^{-3}$. In the simple case of

a monoatomic two-dimensional crystal, c_2 consists of a set of Bragg peaks located at the vertices of the reciprocal lattice.

Supplementary Figure 6 shows the bare dispersion relation of the electrons and the structure factor in the first and second Brillouin zone. The four Bragg peaks corresponding to the second Brillouin zones are located around $\mathbf{k} = (\pm\frac{2\pi}{a}, \pm\frac{2\pi}{a})$. The density of states resulting from the scattering effect is shown on panel c. Copies of the central parabola appear shifted around the Bragg peaks, corresponding to the different Brillouin zones. At the Brillouin zone boundaries, the bands bend and a gap opens. The width of the gap is depends on the scattering strength v_0.

When the atomic lattice is amorphous, $c_2(\mathbf{k})$ consists of one or several concentric rings, resulting from the persistent local order (Supplementary Figure 6). Close to the Brillouin zone boundary, i.e. for $\|a\mathbf{k}\| = \pi$, the resulting band structure is modified compared to the crystalline case. Instead of an avoided crossing, the bands broaden and states appear within the gap (Supplementary Figure 6(b)). These results are consistent with those obtained at the first Born approximation level in [10, 11].

We now generalize this calculation to the case of Dirac electrons at the surface of a topological insulator. The main difference is that the electron dispersion is now made of two distinct bands. The bare Green's function for each band $s = \pm$ reads [12]

$$G_{0s}(E, \mathbf{k}) = \frac{1}{E - s v_{\mathrm{F}} |\mathbf{k}|}. \tag{5}$$

where $\mathbf{k} = k_x, k_y$ is the surface momentum of the electron.

Scattering processes can occur both within a band or between the bands, which results in an additional overlap factor to Eq. (2) [12]

$$F_{ss'}(\mathbf{k}, \mathbf{k}') = \frac{1 + ss' \cos(\theta_{\mathbf{k}} - \theta_{\mathbf{k}'})}{2}, \tag{6}$$

to take into account inter-band scattering effects. With these considerations the self-energy (3) reads

$$\Sigma_s(E, \mathbf{k}) = \frac{v_0^2}{\Omega} \sum_{s'} \int c_2(\mathbf{k} - \mathbf{k}') F_{ss'}(\mathbf{k}, \mathbf{k}') G_{\Sigma s'}(E, \mathbf{k}') \mathrm{d}\mathbf{k}', \tag{7}$$

And the spectral function is then given by

$$\begin{aligned} A(E, \mathbf{k}) &= A^+(E, \mathbf{k}) + A^-(E, \mathbf{k}) & (8) \\ &= -\frac{1}{\pi} \sum_{s=\pm} \mathrm{Im} \left(\frac{1}{E - s v_{\mathrm{F}} |\mathbf{k}| - \Sigma_{s+}(E, \mathbf{k}) - \Sigma_{s-}(E, \mathbf{k})} \right). & (9) \end{aligned}$$

Then, as in the single band case, copies of the free-electron dispersion relation appear at the peaks of the structure factor. But a gap can only open if the two crossing branches belong to the the same band, *i.e.* have overlapping wave vector. In the crystalline case (Supplementary Figure 6(c)), this is not the case. As a consequence, no gap opens

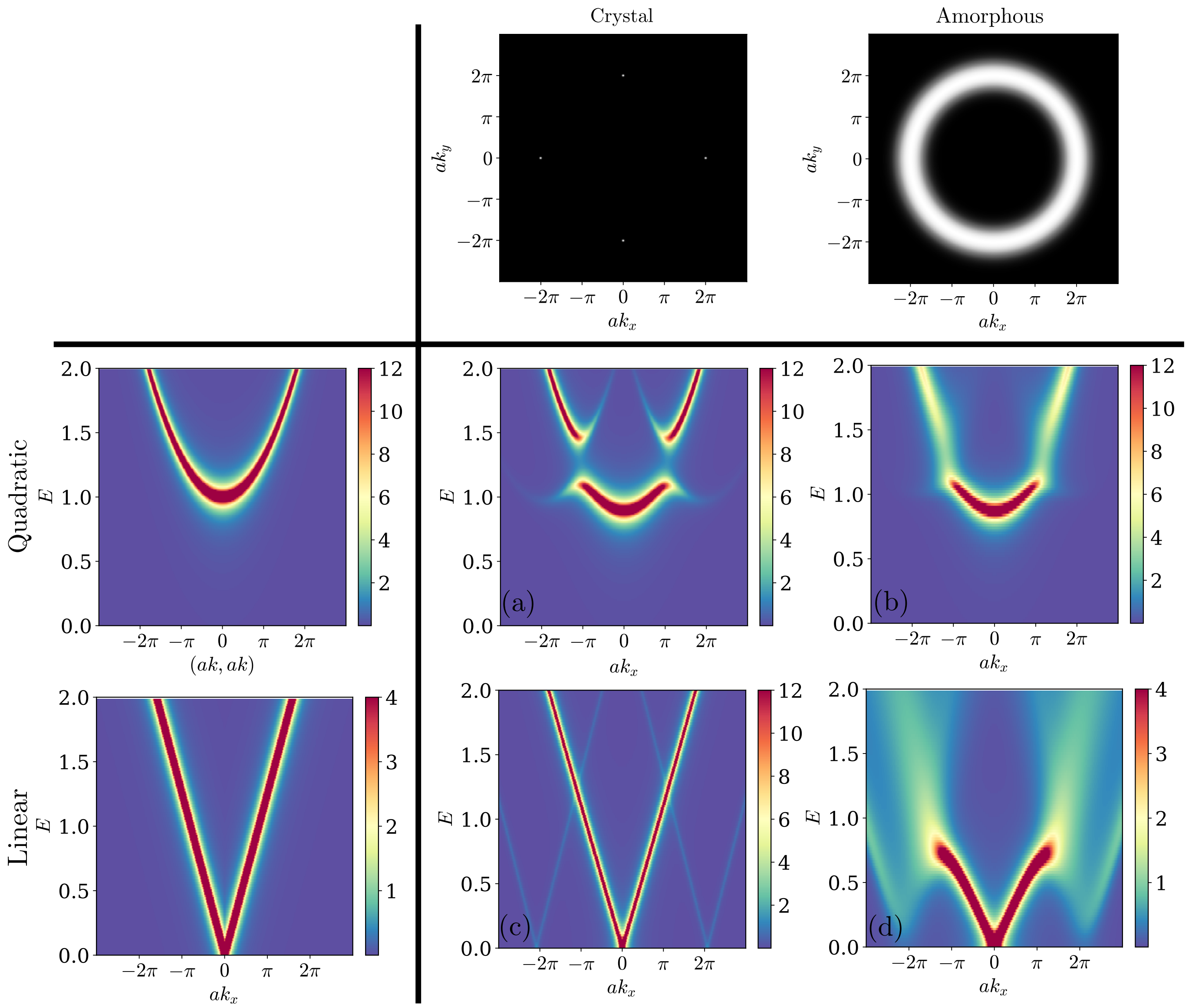

SUPP. FIG. 6. Band structures resulting from massive (top row) and Dirac (bottom row) electrons, interacting with a crystalline (left column) or an amorphous (right column) atomic lattice. (a) In the quadratic case, with a crystalline lattice, copies of the central parabola appear at the center of the neighbouring Brillouin zones. At the Brillouin zone boundary, where two branches cross, the bands bend and a gap opens. (b) In the amorphous case, copies still form in the ring-shaped second Brillouin zone. Around the Brillouin zone boundary, broad vertical features appear within the band gap. (c) In the linear case, the band structure is composed of two bands and interband scattering is forbidden by the overlap factor for anti-parallel momenta. Thus, no gap opens at the Brillouin zone boundary when two branch cross. (d) However, in the amorphous case, the ring-shaped structure factor allows scattering between momenta with arbitrary angles. Then interband scattering process can occur at the Brillouin zone boundary. Similar to the quadratic case, bands bend and states appear within the gap.

when the central Dirac cone and its copies cross at $a\|\mathbf{k}\| = \pi$. On the contrary, in the amorphous case, the isotropic structure factor allows for scattering of non-colinear momenta. Thus, the Dirac cone centered around Γ and the one emerging from the first ring do interact when crossing (Supplementary Figure 6(d)). At the Brillouin zone boundary, the two branches bend and the density of states shows a broad vertical structure, similar to the quadratic band case.

In the main text, the structure factor $c_2(\mathbf{k})$ is reconstructed based on the diffraction pattern experimentally measured on amorphous Bi_2Se_3. Indeed, the orientation-integrated diffraction intensity $I(\mathbf{k})$ is related to the structure factor

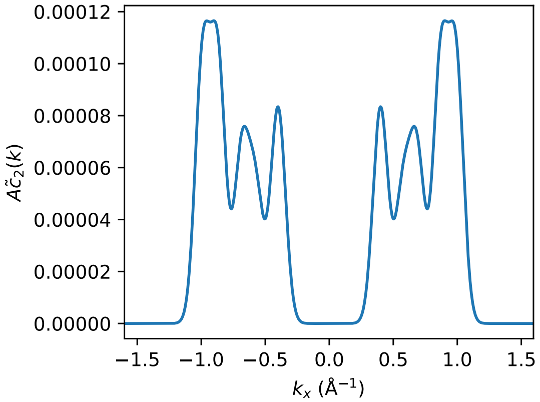

SUPP. FIG. 7. Structure factor used to compute the spectrum (3) in Figure Figure 4(d). The position and relative intensity of the three peaks have been determined from the diffraction pattern in Main Figure 1(c). For this spectrum $A = 1.2 \cdot 10^{-3}$. For the spectrum (2) of the same panel, $A = 0.6 \cdot 10^{-3}$.

$S(q)$ through

$$I(k) = Nf^2(k)\left(1 + \frac{S(k)}{k}\right), \tag{10}$$

where $k = \|\mathbf{k}\|$ and $Nf^2(k)$ is the atomic scattering factor. c_2 used in the calculation is plotted in Supplementary Figure 7.

[1] Rotenberg, E., Theis, W., Horn, K. & Gille, P. Quasicrystalline valence bands in decagonal AlNiCo. *Nature* **406**, 602–605 (2000).

[2] Kresse, G. & Furthmüller, J. Efficient iterative schemes for *ab initio* total-energy calculations using a plane-wave basis set. *Phys. Rev. B* **54**, 11169–11186 (1996).

[3] Kresse, G. & Hafner, J. Ab initio molecular dynamics for liquid metals. *Phys. Rev. B* **48**, 13115 (1993).

[4] Perdew, J. P., Burke, K. & Ernzerhof, M. Generalized gradient approximation made simple. *Phys. Rev. Lett.* **77**, 3865–3868 (1996).

[5] Toh, C.-T. *et al.* Synthesis and properties of free-standing monolayer amorphous carbon. *Nature* **577**, 199–203 (2020).

[6] Torquato, S., Zhang, G. & Stillinger, F. H. Ensemble theory for stealthy hyperuniform disordered ground states. *Phys. Rev. X* **5**, 021020 (2015).

[7] Weiße, A., Wellein, G., Alvermann, A. & Fehske, H. The kernel polynomial method. *Rev. Mod. Phys.* **78**, 275–306 (2006).

[8] Marsal, Q., Varjas, D. & Grushin, A. G. Topological Weaire−Thorpe models of amorphous matter. *Proceedings of the National Academy of Sciences* (2020).

[9] Spring, H., Akhmerov, A. R. & Varjas, D. Amorphous topological phases protected by continuous rotation symmetry. *SciPost Phys.* **11**, 22 (2021).

[10] Edwards, S. F. The electronic structure of disordered systems. *The Philosophical Magazine: A Journal of Theoretical Experimental and Applied Physics* **6**, 617–638 (1961).

[11] Edwards, S. F. & Mott, N. F. The electronic structure of liquid metals. *Proceedings of the Royal Society of London. Series A. Mathematical and Physical Sciences* **267**, 518–540 (1962).

[12] de Juan, F., Hwang, E. H. & Vozmediano, M. A. H. Spectral and optical properties of doped graphene with charged impurities in the self-consistent Born approximation. *Phys. Rev. B* **82**, 245418 (2010).
